# Editorial: Antibody Therapeutics for the Treatment of Filoviral Infection

**DOI:** 10.3389/fimmu.2022.859919

**Published:** 2022-02-18

**Authors:** Charles D. Murin, Bronwyn M. Gunn, Paul W. H. I. Parren, Gary P. Kobinger

**Affiliations:** ^1^ Scripps Research, Department of Integrative Structural and Computational Biology, La Jolla, CA, United States; ^2^ Paul G. Allen School of Global Health, Washington State University, Pullman, WA, United States; ^3^ Department of Immunohematology and Blood Transfusion, Leiden University Medical Center, Leiden, Netherlands; ^4^ Lava Therapeutics, Utrecht, Netherlands; ^5^ Galveston National Lab, Galveston, TX, United States

**Keywords:** antibody therapeutic, ebolavirus, filovirus and viral hemorrhagic fever, vaccine, monoclonal antibodies

A reoccurring theme in human existence is the ever-looming threat of emergent infectious diseases and risk for uncontrolled outbreaks. The unprecedented connectivity in our 21^st^ century society has increased the risk of rapid global spread and we therefore need to effectively leverage our collective knowledge to develop and stockpile effective countermeasures to combat infection and disease more than ever. This notion was brought home by the 2013-2016 Ebola virus outbreak with over 11,000 deaths, which sparked a renewed urgency for the readiness to prevent and treat filovirus infection by vaccines and therapeutics. Antibody therapeutics for filovirus infection matured due to their demonstrated efficacy in the Pamoja Tulinde Maisha trial (PALM; meaning ‘Together Save Lives’ in the Kiswhahili language) conducted in 2018-2019 in the Democratic Republic of Congo. This led to two FDA approvals for the treatment of Ebola virus Zaire infection in adults and children in late 2020. One year prior to that, in 2019, two vaccines for the prevention of Ebola virus disease were approved: Ervebo and the two component Zabdeno/Mvabea vaccine. Despite these advances, new outbreaks of filovirus infections continue to occur and be a threat to human health.

In this Research Topic of *Frontiers in Immunology: Vaccines and Molecular Therapeutics*, we present Original Research articles and Reviews that reflect the current state of antibody therapeutics against filoviral infection and disease, emphasizing the future development of next-generation therapeutics as we learn to combat these viruses. The major themes addressed in this collection include:

1. What are the correlates of protection, particularly for the humoral immune system, in humans exposed to filoviruses?

2. What novel countermeasures are being developed to be ready for the next filovirus outbreak?

3. How can we develop pan-ebolavirus antibody therapeutics and effectively move them forward?

Often the best place to start developing a vaccine or antiviral therapeutic is to first define and understand the immune mechanisms that mitigate disease in natural infection. Paquin-Proulx et al. utilize a large cohort from the Bundibugyo Ebola virus 2007 outbreak in Uganda to show that antibodies with broad effector functions persist in survivors and may lower the likelihood of lingering clinical sequelae, such as hearing loss, that are increasingly being recognized as a long-term complication of filovirus infections. Gilchuk et al. take a proteomic approach detailing the antibody response in a survivor of Ebola virus disease to show that antibodies against the glycan cap and base of the viral glycoprotein are most strongly associated with protection. These studies therefore highlight the importance of focusing on antibodies against certain epitopes of the Ebola virus glycoprotein and incorporating strong antibody-mediated effector activity.

In addition to therapeutic antibodies, vaccines that elicit a robust humoral response are vital to combating viral outbreaks. Earlier, Marzi et al. developed a Marburg virus vaccine that protected non-human primates effectively. Extending their studies, the authors now demonstrate the utility of this vaccine to provide rapid protection, which would be of critical importance during an active outbreak. Complete protection to lethal Marburg virus challenge was achieved 7-14 days post vaccination with, interestingly, innate responses providing partial protection already apparent after 3 days. While intramuscular infection in the non-human primate model has long been used as a stringent model for vaccine and therapeutic protection against lethal infection, one of the limitations of this model has been that the kinetics of disease and progression to death is far more rapid than that observed in human filoviral disease. Johnston et al. demonstrate how intranasal exposure to EBOV more accurately reflects the delayed onset of disease that is seen in humans, including a longer incubation period, and may offer an additional model for future antibody therapeutic and vaccine challenge studies that can be translated to humans.

In the realm of antibody therapeutic treatment, Mbaya et al. review the development of the two therapeutic antibody products: ansuvimab (mAb114; Ebanga) and the tri-mAb cocktail consisting of odesivimab, maftivimab and atoltivimab (REGN-EB3; Inmazeb). The authors discuss the results of the PALM trial and explore ways in which future therapeutics could be tested and improved. Murin et al. review the current state of ebolavirus therapeutics from the structural biology perspective and how it provided insights in viral epitopes that might contribute to pan-ebolavirus responses. Ebola virus cross-species, or cross-filovirus reactivity for that matter, is lacking from the current FDA approved therapeutic antibody products that are Ebola virus Zaire monospecific. The authors speculate how future iterations of antibody therapeutics could take advantage of novel development routes, such as antibody synergy and bispecific antibodies, to maximize potency and cross-reactivity. In addition to ebolaviruses, marburgviruses have caused outbreaks of filovirus disease and therefore should also be considered a continued threat. Previously engineered temporally obligate bispecific antibodies were demonstrated to represent an elegant approach for achieving pan-ebolavirus activity, but a strategy for generating pan-*filoviral* antibodies remained elusive. Wirchnianski et al. expand on their “Trojan horse” method by developing bispecific antibodies that leverage the ubiquitous cation-independent mannose-6-phosphate receptor to deliver an antibody reactive with the filovirus receptor NPC1 into endosomes to prevent entry and provide pan-filoviral protection.

The need for preparedness and strategies to rapidly develop and implement countermeasures to emerging infectious diseases is highlighted by the current COVID-19 pandemic. Strong collaborations of the scientific and medical community with biotech, pharma companies and regulators have shown to be essential to rapidly develop solutions. If we again learned anything, then it is that preparedness is essential. The rapid development of vaccines and therapeutic antibodies during the current COVID-19 pandemic leaned heavily on the lessons learned from tackling filovirus outbreaks. The strategies and novel approaches that have been field-tested for filoviruses, such as those described in this Research Topic, can and should be applied to prepare us for the inevitably next emergent pathogen. The renewed focus on the real and global threat of emerging pathogens highlights the immense value and need for continued investments in researching epidemiology, pathogenic mechanisms and treatments for emergent infections and neglected tropical diseases.

## Author Contributions

CM drafted the manuscript. CM, BG, PP, and GK. edited the manuscript. All authors contributed to the article and approved the submitted version.

## Conflict of Interest

PP is an employee of Lava Therapeutics, a publicly traded biotechnology company that develops therapeutic antibodies including bispecific antibodies and bispecific antibody technology. He obtains stock options as part of his employment.

## Publisher’s Note

All claims expressed in this article are solely those of the authors and do not necessarily represent those of their affiliated organizations, or those of the publisher, the editors and the reviewers. Any product that may be evaluated in this article, or claim that may be made by its manufacturer, is not guaranteed or endorsed by the publisher.

